# Cook County Hospital

**Published:** 1879-07

**Authors:** Jerome H. Salisbury

**Affiliations:** House Surgeon


### Clinical Reports.

ARTICLE IV.

COOK COUNTY HOSPITAL.

(Reported by JEROME H. SALISBURY, M.D., House Surgeon.)

*I. Periodical Mania.*

Case No. 23,205, A. C., was admitted Feb. 3d, 1879; age, 25 years; occupation, a driver; native of the United States.

He gave the following history:

He had been always healthy previous to the present attack, except that he received an injury to the head about eight years ago, from which he recovered perfectly. His habits have been temperate.

Fifteen days ago he came home in the evening earlier than usual, and talked in a strange, inconsistent way. He had three or four scratches on his head, and over the frontal bone was a lump 5 Cm. in diameter.

It was afterwards learned that he had fallen out of his wagon while in motion, but had gotten into it again, and was found under a shed insensible. That night he vomited constantly. About two o'clock a.m., he became rational, and continued so to all appearance for six days. During this time he had some headache, and complained of the noise and the light. He did not vomit after the first night and morning.

On the seventh day he had chills and fever. He lost his reason again at about 11 a.m., regaining it the next morning.

The next two days he had chills and fever, and became maniacal at about 11 a.m., regaining his reason the next morning. After this, until his admission to the hospital, he had a similar attack of mania every day at 11 a.m.

On admission, the patient was maniacal, undertaking to injure all who came near him, and talking wildly and inconsistently, His pupils were moderately dilated. His temperature was 37.8; his pulse strong and full. He was restrained by a straight jacket, and given doses of potassium bromide and hydrate of chloral. Towards evening he became more quiet. He rested quietly from about six to nine p.m., when he became restless and maniacal. Another dose of bromide and chloral was given, and he rested fairly well during the night.

The next morning, Feb. 4th, the patient was quiet. On account of the intermittency of the attacks, he received one half gram of sulphate of quinia at about 9 a.m. A dose of bromide and chloral was also given. The paroxysm of mania did not occur. In the evening the patient was quiet. He had a little headache, ate well, and his bowels moved.

Feb. 5th and 6th he received 25 centigrams of sulphate of quinia, and no paroxysm occurred. On the night of the 6th he was somewhat delirious, but not violent.

He was quiet all day on the 7th, but was not rational. The next day he became rational, and continued so. He improved in every respect until the 14th, when he was discharged recovered.

When last heard from, the patient was pursuing his business and was well, with the exception that he had some pain in the head whenever he became excited.

*II. Cancer of Gall Bladder and Multiple Abscess of the Liver.*

Case No. 23,264, M. McC., age 63; occupation, a janitor; born in Scotland. Admitted to the hospital Feb. 13th, 1879.

Patient's family history was good. At the age of 17, the patient had intermittent fever. At the age of 20 he had, while in South America, an attack of dysentery lasting about three weeks. At the age of 40 he had, while in India, a low form of fever prevalent in that country, which lasted about six weeks, the convalescence from which occurred in eight months. Since this time his health has been excellent. His habits have been temperate.

About two weeks previous to admission, he began to have severe headache and fever. The next evening he had a chill and a pain in the right lumbar region. The third evening he had another chill, with pain extending around to the front.

From that time until admission he had no chills, but the pain continued, with slight remissions and exacerbations. During this time his appetite was poor. His bowels moved once in the two weeks. He had no nausea nor vomiting.

On admission, the patient was seen to be fairly nourished. His pulse and respirations were normal. His tongue was large, moist and flabby. His appetite was poor, and his bowels had not moved for six days.

The liver was found to be somewhat enlarged and tender. The urine was : sp. g. 1028 — reddish yellow, neutral — no albumen, no bile. The urine was examined on the two succeeding days with the same result.

The conjunctivæ were somewhat yellow, but the jaundice was not very marked. February 17th, the jaundice was more marked and continued to increase until February 25th, when it became very marked, the skin being quite yellow. From this time until death, this symptom continued with slight fluctuations. February 23d, the urine gave a reaction for bile. Constipation and tenderness over the region of the liver were constant symptoms throughout the course of the disease. About two weeks after admission, enlargement of the veins over the region of the liver was noticed.

March 5th he had a chill and had chills at irregular intervals of two or three days until his death. His appetite became still poorer, and he emaciated greatly. His sleep was very poor. He had frequently pain in the lower part of the abdomen. He sweat profusely at night. He grew weaker gradually and died May 4th. His temperature for the last two months ranged from 97–99 in the morning, and from 100 to 103 in the evening.

Autopsy : Heart, lungs, spleen and kidneys, normal. The liver was very much enlarged and of a dark brown color. The extremity of the gall bladder and the surrounding tissue were the seat of a cancerous growth. The gall bladder was filled with a green, watery fluid, containing a number of gall stones. In the right lobe of the liver near its outer margin, were two secondary cancerous masses of the size of a walnut.

On section of the liver the hepatic ducts were found much dilated, and in their walls numerous small abscesses were found. Microscopical examination showed the cancer to be a cylindrical epithelioma, and the secondary masses were of the same character, rendering it probable that the cancer germs had traveled up the bile ducts to reach the spot where the secondary masses were deposited.

*III. Stricture of Trachea.*

No. 23,419, Matilda F., age, 31; occupation, housewife; native of Germany; admitted March 17th, 1879; family history, good. Her previous health was good until a year ago, when she took cold and began to cough. Her cough increased in severity, and also the quantity of expectorated matter until admission, being aggravated by fresh "colds." Two weeks before admission, she took to her bed, and had chills nearly every day until admission. On admission, the patient was poorly nourished and emaciated. She stated that the emaciation had come on since Christmas. Her appetite was lacking, but she had great thirst. Her tongue had a slight dirty yellow coat. She had some looseness of the bowels. She coughed a good deal and expectorated an abundant thick white sputum. She had considerable dyspnœa. She had night sweats. She had no pain anywhere.

Examination showed the percussion note good over the entire chest. Expiratory murmur prolonged both in front and behind. Dry and moist râles were heard scattered over the chest. The vocal resonance was good. The diagnosis made was bronchitis and asthma.

March 24th she had a chill; March 26th she had a chill. March 29th, a whistling murmur heard with the first sound of the heart, and heard only on the right side of the sternum, was found.

April 9th, the râles had disappeared. April 16th, had two chills. Her appetite at this time was good. On the 29th of April, she was feeling well. May 4th, she died suddenly with great dyspnœa.

Autopsy, 36 hours after death; heart, brain, liver and spleen normal. Some chronic gastric catarrh. Lungs: old pleural adhesions were found. There was found a catarrhal pheumonia of the anterior and inferior portion of both lungs; also a bronchitis of the larger as well as the smaller bronchial tubes. Just above the bifurcation of the trachea was found a fusiform stricture about an inch in length, and narrowing the caliber of the trachea to about a line in diameter. The stricture was in an ulcerated condition. Death was probably due to the blocking up of the narrowed trachea with a plug of mucus.

